# A Novel Concentrated, Interdisciplinary Group Rehabilitation Program for Patients With Chronic Obstructive Pulmonary Disease: Protocol for a Nonrandomized Clinical Intervention Study

**DOI:** 10.2196/40700

**Published:** 2022-10-26

**Authors:** Bente Frisk, Kiri Lovise Njøten, Bernt Aarli, Sigurd William Hystad, Sidsel Rykken, Ane Kjosås, Eirik Søfteland, Gerd Kvale

**Affiliations:** 1 Department of Health and Functioning Western Norway University of Applied Sciences Bergen Norway; 2 Helse i Hardanger Øystese Norway; 3 Department of Thoracic Medicine Haukeland University Hospital Bergen Norway; 4 Department of Clinical Science University of Bergen Bergen Norway; 5 Department of Psychosocial Science University of Bergen Bergen Norway; 6 Department of Medicine Haukeland University Hospital Bergen Norway; 7 Division of Psychiatry Haukeland University Hospital Bergen Norway; 8 Department of Clinical Psychology University of Bergen Bergen Norway

**Keywords:** COPD, pulmonary rehabilitation, chronic illness, interdisciplinary, chronic disease, rehabilitation model, rehabilitation, treatment, group therapy, patient outcome, health intervention, pulmonary disease, intervention study

## Abstract

**Background:**

Pulmonary rehabilitation has been demonstrated to be a highly effective treatment for people with chronic obstructive pulmonary disease (COPD). However, its availability is scarce worldwide, and new and innovative rehabilitation models are highly warranted. Recently, the group behind the present study published a protocol describing a novel concentrated, interdisciplinary group rehabilitation program for patients with chronic illnesses. The current paper describes an extension of this protocol to patients with COPD.

**Objective:**

The objective of this study is to explore the acceptability of concentrated, interdisciplinary group pulmonary rehabilitation for patients with COPD. The intervention is expected to improve functional status and be highly acceptable to patients.

**Methods:**

This study will include 50 patients aged over 40 years who fulfill the diagnostic criteria for COPD: a forced expiratory volume at the first second (FEV_1_) <80% of expected and a FEV_1_/forced vital capacity ratio below the lower limit of normal according to the Global Lung Function Initiative. An interdisciplinary team consisting of physicians, physiotherapists, psychologists, pharmacists, clinical nutritionists, and nurses will deliver the treatment to groups of 6 to 10 patients over 3 to 4 consecutive days with a 12-month follow-up. The intervention is divided into three distinct phases: (1) pretreatment preparation for change, (2) concentrated rehabilitation, where the patient is coached to focus on making health-promoting microchoices, and (3) integration of the changes into everyday living, aided by digital follow-up and 2 on-site clinical examinations. Statistical significance will be set at α=.05.

**Results:**

The recruitment period will last from April 2022 until June 2023.

**Conclusions:**

If successful, this highly novel rehabilitation format might change the way we deliver care for patients with COPD, leading to substantial societal and socioeconomic gains. The study will expand knowledge on the concentrated treatment format as a rehabilitation model for people with COPD.

**Trial Registration:**

ClinicalTrials.gov NCT05234281; https://clinicaltrials.gov/ct2/show/NCT05234281

**International Registered Report Identifier (IRRID):**

PRR1-10.2196/40700

## Introduction

Chronic obstructive pulmonary disease (COPD) is characterized by irreversible airflow limitation [1], with dyspnea, cough with sputum production, and fatigue as the main symptoms [1]. The disease is the third leading cause of death worldwide [2], and in 2019 3.23 million deaths were caused by COPD [2]. COPD represents a global health challenge and causes major economic and societal burdens [3,4].

Guidelines highlight that pulmonary rehabilitation is the most important treatment option in the integrated care of patients with COPD, and it ranks as one of the most cost-effective treatment strategies [1,5]. Clinically meaningful improvements in exercise tolerance, dyspnea, fatigue, anxiety and depression, lower-limb muscle strength, self-efficacy, and health-related quality of life have been demonstrated after participation in pulmonary rehabilitation, irrespective of the baseline clinical status [6]. Despite these documented effects, the treatment is underused worldwide. Data from the United States and Canada demonstrate that less than 5% of eligible patients ever participate in a pulmonary rehabilitation program [7,8], and a recently published study from Norway showed that only 5% of Norwegian municipalities had multidisciplinary pulmonary rehabilitation programs for patients with COPD [9]. The underuse of pulmonary rehabilitation calls for new rehabilitation models to increase accessibility and personalization to address individual patient goals, which is believed to improve patient outcomes [10].

Based on extensive experience with the concentrated rehabilitation format [11-15], the group behind this project recently published a protocol for concentrated, interdisciplinary group rehabilitation for chronic illnesses [16]. So far, patients with chronic low back pain, post–Covid-19 symptoms, anxiety and depression, and type 2 diabetes have been included. These disorders were chosen because they each represent a major societal challenge, and novel, effective, and cost-effective treatment approaches are highly needed. The primary symptoms of the included disorders are highly diverse, but they share health challenges that can either improve or worsen depending upon patients’ own handling of their symptoms. By including patients with COPD, we may be able to further increase our knowledge of concentrated rehabilitation models for these patients. Existing rehabilitation models for patients with COPD usually have a duration of 6 weeks or more for outpatient models and 3 to 4 weeks for inpatient models [17]. A concentrated intervention period could increase access to rehabilitation and thereby increase availability.

Uncertainties related to their prognosis, health-related worries, and rumination are common in people living with COPD [18]. Most patients know the importance of being physically active, sleeping well, eating healthily, and taking their medication as prescribed, but are often unable to make use of general health advice, as their primary concern is avoiding worsening of their symptoms. However, a focus on symptom regulation with the intention of preventing worsening might actually risk increasing symptoms by conserving the health problem, particularly if the first indication of improvement is a temporary worsening of symptoms, such as dyspnea or tiredness after increased physical activity. Breaking this unhelpful pattern of symptom regulation is at the core of the concentrated rehabilitation model.

The overall structure of the concentrated rehabilitation model is identical across all disorders [16]. Aided by an interdisciplinary team, patients work in a safe setting to use current coping strategies that are described in the published protocols for chronic illnesses [16]. Our rehabilitation program can be summarized into three stages: (1) pretreatment, in which patients are prepared for change by thoroughly introducing them to the details of the rehabilitation program before it starts; (2) concentrated rehabilitation, which is delivered over 3 to 4 consecutive days in groups of 6 to 10 patients; and (3) follow-up, which includes daily digital follow-ups for the first 3 weeks after the intervention, followed by questionnaires and physical examinations at 3, 6, and 12 months. A web app will be used for the digital follow-up and for the questionnaires.

During the concentrated intervention, each patient is assisted by the interdisciplinary team, which includes physicians, physiotherapists, psychologists, pharmacists, clinical nutritionists, and nurses. This team helps patients explore new approaches in how to deal with their symptoms. Specifically, the patients are instructed to view each symptom as an opportunity to break unhelpful patterns of symptom regulation by doing things differently (ie, making “microchoices”). This approach enables the patients to systematically increase their flexibility and level of functioning when symptoms and health challenges are present. All patients focus on breaking unhelpful patterns of symptom regulation that are relevant to themselves. In addition, focusing on deliberate behavior and action instead of symptoms implies that change is within reach and possible to control [16]. Each day, the group attends a joint program for approximately 8 hours, and participants then attend individually planned training sessions in the afternoon and evening. The long sessions give each patient the opportunity to practice a broad range of potential microchoices. The training is interspersed with brief group sessions, in which each patient shares their progress in targeting and breaking unhelpful patterns of symptom regulation and receives expert feedback on how to improve their outcome by considering all microchoices. The group format offers substantial opportunities for both inspiration and support. On the last day of the intervention, every patient makes an individualized plan on how to integrate the changes they have learned into everyday living, summarized under the headings “Eat,” “Move,” and “Sleep,” and how to systematically make microchoices consistent with increased everyday functioning.

The aim of this study is to explore the acceptability of concentrated, interdisciplinary group rehabilitation for patients with COPD. The intervention is expected to improve functional status and to be highly acceptable to patients.

The main hypotheses are identical to the ones previously published for other disorders [16].

We hypothesize that the treatment will be acceptable, as indicated by the following: the proportion of patients who meet the inclusion criteria and accept participation will be >90%; the proportion of included patients who attend the concentrated intervention will be >90%; and the proportion of included patients who complete participation in the concentrated intervention will be >90%.

Further, we hypothesize that the patients will increase their functional exercise capacity, as measured by functional performance tests that include the Stair Climbing Test and the 60-Second Sit-to-Stand Test; improve their health status 3, 6, and 12 months after the intervention, as measured by the COPD assessment test (CAT) [19]; and be satisfied with the treatment, as defined by a mean Client Satisfaction Questionnaire 8 (CSQ-8) [20] score of 20 or more, with no single dimension below an average score ≤2. A cut-off score of 20 was chosen based on previous research that used scores ≥20 to indicate “good” satisfaction [21].

Finally, we hypothesize that there will be a significant change in the extent to which COPD affects the patients’ lives, as measured with the Brief Illness Perception Questionnaire (BIPQ) [22] with the following questions: “How much does your illness affect your life?” “How much control do you feel you have over your illness?” “How concerned are you about your illness?” and “How well do you feel you understand your illness?”

## Methods

### Overview

The study is part of the Project Development of Smarter Health Solutions (PUSH) project, which is a collaboration between Haukeland University Hospital (Bergen, Norway) and Helse i Hardanger (HiH) (Øystese, Norway). The treatment is delivered at HiH in Øystese, which is a small municipality outside Bergen. The methods for this open, nonrandomized study are identical to already published protocols for low back pain, type 2 diabetes, post–COVID-19 fatigue, and depression or anxiety [16].

### Study Design and Participants

This is a nonrandomized, 1-group pre-post study with a longitudinal 12-month follow-up period and the intention to test the acceptability and effectiveness of an interdisciplinary, concentrated group rehabilitation program for patients with COPD.

We will recruit 50 patients fulfilling the following inclusion criteria: motivation for change regarding self-management of COPD and a diagnosis of COPD based on a postbronchodilation forced expiratory volume in 1 sec (FEV_1_) to forced vital capacity (FVC) ratio below the lower limit of normal according to the Global Lung Function Initiative [23]. The patients must be fluent in oral and written Norwegian, have access to a smartphone, and have sufficient digital competence to handle online questionnaires. They must be independent in activities of daily living, able to perform physical activities indoors and outdoors, and able to climb stairs. Patients will not be excluded if there are exacerbations requiring medical treatment during the last 4 weeks prior to inclusion, but their inclusion will be deferred. Patients with inflammatory disorders that affect activity level, such as rheumatoid arthritis, systemic lupus erythematosus, or other connective tissue disorders; inflammatory bowel disease; or any active cancer in the last 5 years will be excluded. Common chronic diseases with known inflammatory components, such as chronic heart disease, diabetes, and hypertension, will not be a cause for exclusion.

The treatment will be delivered in groups of 6 to 10 patients. The dates for the group sessions are predecided, and patients will be successively allocated to the groups upon availability.

### Procedures and Patient Flow

**Figure 1:**
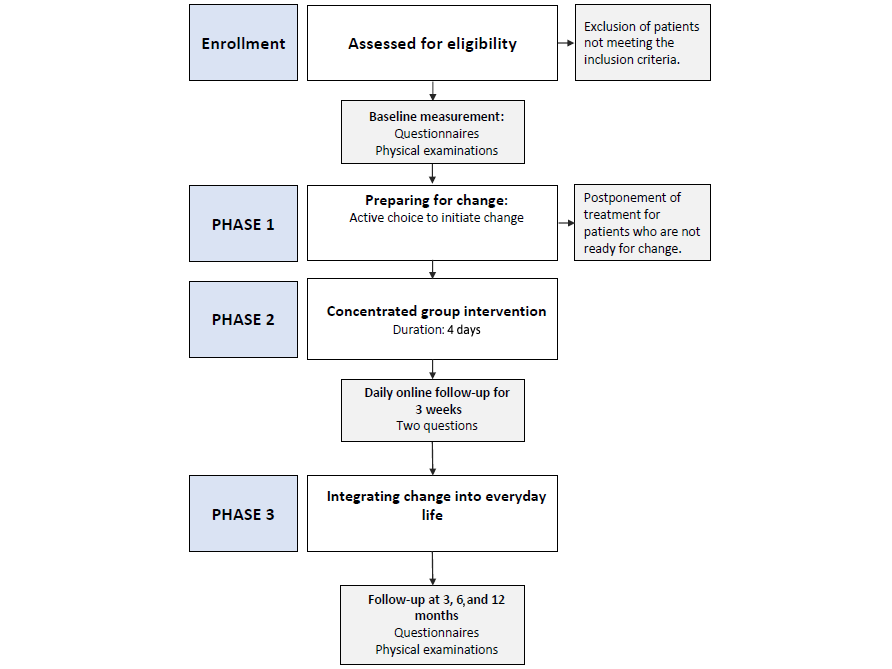
Flowchart of the study. Physical examinations at baseline and the 3-, 6-, and 12-month follow-ups include a cardiopulmonary exercise test, the Stair Climbing Test, the 60-Second Sit-to-Stand Test, and a lung function test. The cardiopulmonary exercise test will be administered only at baseline and at the 12-month follow-up. Daily online follow-up (in phase 2) includes two questions: (1) “To what extent did you allow the symptoms to decide today?” and (2) “To what extent did you make use of the principle of ‘doing something else’?” (responses range from 0-10 for both).

Patients will be referred by their general practitioners or other physicians responsible for their treatment to the PUSH project at the Department of Thoracic Medicine at Haukeland University Hospital. To be included, the patients must be entitled to health care in the specialist health service. Patients who, based on the referral, seem to fulfill the inclusion criteria and none of the exclusion criteria will be called by one of the clinicians and will receive information about the PUSH project and the concentrated rehabilitation (Figure 1). If they are in the target group, an appointment for screening will be made. To ensure all participants receive identical information about the program, they will be asked to watch a video describing the program. The informed consent form will be signed online prior to answering the questionnaires. Each patient will then be invited to a physical examination where lung function and exercise capacity will be assessed. To ensure that the patients are prepared and ready for the intervention, they will be invited to a 1-hour group meeting 1 to 3 weeks prior to the concentrated rehabilitation, where details of the program will be described. The week before the intervention, the group leader will call each patient to ensure that they have received the necessary information and are ready for the concentrated rehabilitation program.

### Outcome Measures

The outcomes presented in this protocol paper address the patients’ overall experiences with the concept of the concentrated treatment format. These measures will be assessed before the start of the program and 1 week, 3 months, 6 months, and 12 months after the program. Assessments of exercise capacity will be done before and at 3-month, 6-month, and 12-month postrehabilitation follow-ups. Initial results will be published based on the 3-month follow-up data and final results will be published based on the complete intervention, including the 12-month follow-up data.

### Primary Outcome Measures

The acceptability of the concentrated rehabilitation program will be measured by the following variables: (1) the proportion of patients agreeing to participate in the rehabilitation among those fulfilling the inclusion criteria and offered participation; (2) the proportion of patients offered participation that start treatment; and (3) the proportion of patients that complete the treatment program (on-site). Acceptability will be measured before the start of the rehabilitation program, and during the intervention, completers and noncompleters will be registered.

The CSQ-8 [20] is an 8-item questionnaire that measures patient satisfaction with health services, where the items are rated from 1 (very low satisfaction) to 4 (very high satisfaction). The total score ranges from 8 to 32, with higher scores indicating a higher degree of satisfaction. The CSQ-8 has good psychometric properties, high internal consistency (Cronbach α=.93), and high interitem correlation [24]. The CSQ-8 will be administered 1 week after the intervention.

The BIPQ is a 9-item questionnaire designed to assess cognitive and emotional representations of illness [22]. Questions are graded from 1 to 10. The last item deals with perceived causes of illness; respondents list the perceived 3 most important causal factors in their illness. For this questionnaire, the general word “illness” can be replaced by the name of a particular illness. The word “treatment” in the treatment control item can be replaced by a particular treatment, such as “surgery” or “physiotherapy.” The scale has good psychometric properties according to a recent review [25]. The BIPQ will be administered at baseline and 1 week, 3 months, 6 months, and 12 months after rehabilitation.

### Secondary Outcome Measures

Pre- and posttreatment and at the follow-up assessments at 3, 6, and 12 months, the patients will be asked to rate on a scale from 0 to 10 the extent to which they used the following strategies when trying to handle their symptoms. This questionnaire was developed in cooperation with patients with previous experience in the concentrated treatment format.

1. Wait to start an activity until I feel up to it
2. Wait to start an activity until I am certain that I will succeed
3. Ensure that the symptoms will not get worse
4. Ensure that I am prepared to handle challenges
5. Try to calm down before proceeding when I get anxious
6. Spend a lot of time on worrying and ruminating
7. Avoid socializing if I do not feel up to it
8. Ensure that I get enough rest
9. Try to not let others see how I feel
10. Try to have a positive mindset
11. Follow my gut feeling

### Evaluations at Baseline and 3- and 12-Month Follow-Ups

Height and body mass will be measured to an accuracy of 0.5 cm and 0.1 kg, respectively. The patients will undergo bioelectrical impedance measurements of fat-free mass and fat mass after an overnight fast (InBody 770). BMI will be calculated by dividing weight by the square of height (kg/m^2^), fat-free mass index as the fat-free mass divided by the square of height, and the fat-mass index as the fat mass divided by the square of height [26].

Spirometry will be conducted on a Vyntus Body/Aerosol Provocation System Plethysmograph (Vyaire Medical GmbH) according to the American Thoracic Society/European Respiratory Society standardization of lung function testing [27]. The highest FEV_1_ and FVC ratio values from at least 3 satisfactory expiratory maneuvers will be used. Maximal voluntary ventilation will be directly measured by breathing as deeply and frequently as possible for 12 seconds in a standing position.

Cardiopulmonary exercise tests (CPETs) will be performed on a treadmill with a gradually increasing incline until exhaustion (Woodway). Prior to each CPET, complete volume and gas calibration will be done. All patients will receive initial training to become familiar with treadmill walking before the test starts. The test will start with 3 minutes of warm-up, and steady-state measurements will be conducted with a walking speed individually set between 1.8 and 3.8 kilometers per hour and initial inclination set at 0% based on the predicted fitness level of the patient. The inclination will be increased every 60 seconds by 2%, finally reaching 20%. If the participants can still continue, the speed will increase by 0.5 kilometers per hour until the patient reaches exhaustion. The test will be terminated if the test subject is unable to continue (even with encouragement), if they experience pronounced pain or dizziness, if there are ischemic electrocardiogram (ECG) changes, or if there is a decrease in systolic pressure below the resting pressure [28]. Gas exchange and ventilatory variables will be measured continuously with breath-by-breath sampling, averaged over 30-second intervals through a Hans Rudolph 2-way breathing mask (V2 Mask). The breathing mask will be connected to a Vmax SensorMedics metabolic analyzer (Jaeger CPX Vyntus) to assess ventilatory variables and the content of oxygen and carbon dioxide of expired air to calculate oxygen uptake. Percutaneous oxygen saturation (SpO_2_) will be measured with an ear probe using a stationary pulse oximeter. Heart rate will be measured through a 12-lead ECG (Custo Cardio 300; Custo Med). Blood pressure will be measured with a Tango M2 (SunTech Medical). Ratings for dyspnea and leg fatigue will be recorded every second minute throughout the test using the Borg Category-Ratio 10 scale [29], with a final rating during maximal effort at the end of the test. All assessments will be measured at rest in a sitting position before the start of the test, throughout the exercise test, and for 2 minutes after termination of the test.

The Stair Climbing Test will be used to assess submaximal exercise capacity and will be performed as standardized by Tveter et al [30]. The participants walk up and down 18 steps 3 times as fast as possible. They can walk or run but not skip any steps. Time in minutes and seconds is the main outcome of the test.

The 60-Second Sit-to-Stand-Test will be used to assess lower extremity strength and will be performed as standardized by Tveter et al [30]. The participants start in a seated position in a chair with arms crossed and raised 45 cm, without any armrest, and will be instructed to complete as many full stands as possible in 60 seconds. The number of repetitions is the main outcome.

The CAT will be used to measure the impact of COPD in daily life. The questionnaire consists of 8 questions; possible scores range from 0 to 40 [19,31]. A score below 10 indicates a low impact of symptoms, a score between 10 and 20 medium impact, a score between 21 and 30 high impact, and a score over 30 very high impact [32]. A minimum clinically important change is a reduction of between 2 to 3 points [33]. The following are other measures that we will use, with their respective purposes: The Modified Medical Research Council scale is a self-administered grading system used to measure symptoms of dyspnea; the scale ranges from 0 to 4, with higher scores indicating more severe symptoms [34]. The Dyspnea-12 scale is a patient-reported scale for measuring the severity of breathlessness consisting of 12 descriptors to cover physical and psychological dimensions [35]. The General Anxiety Disorder-7 scale assesses generalized anxiety disorder [36]. The Patient Health Questionnaire-9 is a self-reported questionnaire for screening, diagnosing, monitoring, and measuring the severity of depression [37]. The Bergen Insomnia Scale measures insomnia, consisting of 6 items, with the first 3 pertaining to sleep onset and the last 3 referring to not feeling adequately rested [38]. The International Physical Activity Questionnaire–Short Form measures daily physical activity [39]. The Nordic Musculoskeletal Questionnaire quantifies musculoskeletal pain in 9 body regions [40]. The Strength, Assistance With Walking, Rising From a Chair, Climbing Stairs, and Falls questionnaire is a screening tool to identify patients with probable sarcopenia [41].

### Intervention

The protocol paper reports details of the intervention [16]. In summary, the intervention consists of 3 phases (shown in Figure 1): (1) preparing for change, (2) the concentrated intervention lasting for 3-4 days, and (3) integrating change into everyday living [16]. The focus throughout the intervention is on how to initiate and maintain change by breaking inflexible patterns of symptom regulation.

### Statistical Analyses and Data Handling

The data will be analyzed with SPSS (version 28; IBM Corp) and Stata (version 17; Stata Corp). Changes in self-reported symptoms, level of everyday functioning, and exercise capacity will be examined with repeated-measure analyses. Statistical significance will be set at α=.05. The Glass delta, with pretreatment SD as the denominator, will be used to calculate within-group effect sizes. The Glass delta is the recommended effect size for intervention studies, because there are reasons to believe that the treatment will influence SD, as well as the mean [16]. Effect sizes are usually interpreted as small (0.2), moderate (0.5), or large (0.8). Considering that the current research is a nonrandomized study of a novel, interdisciplinary group treatment for patients with COPD, we expect small to moderate effect sizes.

The number of participants needed to be included in a pragmatic nonrandomized study varies in the literature. However, according to the central limit theorem, the mean of a sample of data will be closer to the overall mean for the population who are studied when the sample size increases [42]. As a rule, sample sizes around 30 to 50 are suggested to be sufficient for the central limit theorem to hold, meaning that the sample means have a normal distribution. We decided to include 50 participants to enable long-term follow-up of the patients.

Missing data regarding the primary outcome variables will be handled by multiple imputation (MI). Under the missing at random (MAR) assumption, MI is presently one of the best available methods of dealing with missing data and will provide unbiased estimates [16]. The main analyses of the primary outcomes will thus be completed under the MAR assumption. Sensitivity analyses will be performed to evaluate the robustness of the results and the potential impact that nonignorable missing data could have on the predicted results. These sensitivity analyses will be based on the pattern-mixture model [16]. In short, a pattern-mixture model involves assumptions that participants lost to follow-up can have a mean outcome that differs by an offset from participants who do not drop out. The impact on the results of various choices of clinically possible offsets can then be examined, and if the effect from the primary analysis is qualitatively maintained for the range of plausible offsets, the findings can be assumed to be robust.

### Data Collection and Monitoring

The data will be collected electronically and by physical examination, and all sensitive data will be stored on an encrypted server at Western Norway Regional Health Authority Information and Communication Technology. Once the patients are included, all data entered by the participants will be monitored by an established study administrative team.

### Adverse Events

If an acute condition occurs, patients will receive the necessary care and might be excluded from the study if there are concerns about safety. Such patients will be thoroughly described and accounted for in line with the illness-specific standard operating procedures. Acute exacerbations during the intervention period will be registered.

### User Involvement

Helse i Hardanger has established a broad user panel with representatives recruited through Haukeland University Hospital and Helse i Hardanger. The following organizations are represented: Norwegian Asthma and Allergy Association, Norwegian Rheumatics’ Association, Mental Health Norway, Breast Cancer Association, Norwegian Diabetes Association, Norwegian Association for Lung and Heart Disease, and Grannehjelpa (“neighbors’ help” in Norwegian). The panel has given feedback throughout the development of the protocol and approved the final version.

### Ethics Approval

The PUSH project and the web app have been approved by the Western Norway Regional Committees for Medical and Health Research Ethics (REK 2020/101638). The same organization approved this study (REK 2021/219567). The study was registered at ClinicalTrials.gov (NCT05234281). Written consent will be obtained from all participants prior to study participation, and the project will be conducted in accordance with the Helsinki Declaration.

### Gender Perspectives

The inclusion criteria, as well as all interventions, are gender neutral. However, to ensure adequate external validity and proper representation, we have no absolute limits in terms of minimum inclusion rates of one gender.

## Results

Recruitment started in April 2022. For the initial 3-month results, recruitment is expected to be completed by June 2023.

## Discussion

### Principal Findings

In this paper, we describe a protocol for the establishment and initial evaluation of a concentrated, interdisciplinary group rehabilitation program for patients with COPD. We will explore the acceptability of the intervention and hypothesize that the intervention will be positively accepted by the participants, will reduce the impact of the illness on their lives, and will improve their level of daily functioning.

This is an extension of a protocol that we recently published for a novel concentrated, transdiagnostic group intervention for patients with chronic low back pain, post-Covid-19 symptoms, anxiety or depression, or type 2 diabetes [16].

Existing rehabilitation programs often have a duration of 4 to 12 weeks, but there is no consensus about the optimal duration [17]. However, longer programs are thought to enable greater gains and allow the maintenance of benefits, with 8 weeks being considered the minimum to achieve a clinically meaningful change [16,43,44]. The future of pulmonary rehabilitation will rely on rehabilitation models that include more choices for the patient and the possibility of increased personalization of the program [10]. If the concentrated pulmonary rehabilitation program is accepted by the patients in this study and leads to an enhanced functional status, large groups of patients might benefit from it in the future. Each patient will receive a tailored program in a group setting, which will provide the opportunity to utilize the group effect in addition to individualizing the treatment to the patient’s own goals and challenges.

This is an open, nonrandomized study, with the participants as their own controls (in a pre-post comparison) and 12-month follow-up period. Although this allows summarizing the experiences and findings as described, it does not allow drawing causal conclusions on the effects of the intervention. The protocol describes a concentrated rehabilitation approach. If the intervention appears to lead to an improvement in health, it could trigger follow-up studies investigating the effects of this intervention in comparison to traditional ways of delivering pulmonary rehabilitation [6].

### Limitations

Drop outs and poor adherence to the intervention might threaten the internal validity of the study. Although we aim to give clear information to the participants to limit this problem, we cannot be sure that they will participate for the whole period or that they will complete the digital and clinical examinations at the 3-, 6-, and 12-month follow-ups. A notice will be sent to the patients approximately 2 to 3 weeks before each assessment. Participants who do not answer the questionnaires online or do not show up for clinical follow-ups will be contacted by phone. The impact of missing data will be evaluated with appropriate statistical methods. Finally, although the acceptability of the concentrated treatment format has been validated for persons with diseases other than COPD [15,45], the outcomes have not been formally validated. To compensate for this, all reasons for not accepting, attending, or completing the intervention will be recorded.

### Conclusion

We present a protocol for the establishment and evaluation of a concentrated pulmonary rehabilitation program for patients with COPD. The treatment focuses on how to initiate and maintain change, with a shift away from monitoring symptoms toward an active approach to daily health-promoting microchoices. This concentrated intervention has the potential to change the way we deliver pulmonary rehabilitation to patients with COPD and might increase the availability of pulmonary rehabilitation to patients who would benefit from this treatment. This rehabilitation model might be a useful addition to the treatment armamentarium of the health care system in the face of coming sociodemographic challenges, and might also increase the availability of pulmonary rehabilitation to the public.

## Abbreviations

BIPQ: Brief Illness Perception Questionnaire
CAT: Chronic Obstructive Pulmonary Disease Assessment Test
COPD: chronic obstructive pulmonary disease
CPET: Cardiopulmonary Exercise Test
CSQ: Client Satisfaction Questionnaire
ECG: electrocardiogram
FEV1: forced expiratory volume at the first second
FVC: forced vital capacity
HiH: Helse i Hardanger
MAR: missing at random
MI: multiple imputation
PUSH: Project Development of Smarter Health Solutions

## Data Availability

In accordance with the approvals granted for this study by the Regional Committee on Medical Research Ethics and the Norwegian Data Inspectorate, the data files will be stored securely and in accordance with the Norwegian Law of Privacy Protection. A subset of the data file with anonymized data will be made available to interested researchers upon reasonable request to author BF, providing that Norwegian privacy legislation and the General Data Protection Regulation are respected and that permission is granted by the Norwegian Data Inspectorate and the data protection officer at Haukeland University Hospital.
